# Dual-layer dual-energy CT-derived pulmonary perfusion for the differentiation of acute pulmonary embolism and chronic thromboembolic pulmonary hypertension

**DOI:** 10.1007/s00330-023-10337-4

**Published:** 2023-11-03

**Authors:** Roman Johannes Gertz, Felix Gerhardt, Michael Pienn, Simon Lennartz, Jan Robert Kröger, Liliana Caldeira, Lenhard Pennig, Thomas Henning Schömig, Nils Große Hokamp, David Maintz, Stephan Rosenkranz, Alexander Christian Bunck

**Affiliations:** 1 Department of Radiology, Faculty of Medicine and University Hospital Cologne, University of Cologne, Cologne, Germany; 2 Department of Cardiology, Heart Center, Faculty of Medicine, University of Cologne, Cologne, Germany; 3 Ludwig Boltzmann Institute for Lung Vascular Research, Graz, Austria; 4 Department of Radiology, Neuroradiology and Nuclear Medicine, Ruhr University Bochum, Johannes Wesling University Hospital, Bochum, Germany

**Keywords:** Computed tomography, Pulmonary perfusion, Acute pulmonary embolism, Chronic thromboembolic pulmonary hypertension

## Abstract

**Objectives:**

To evaluate dual-layer dual-energy computed tomography (dlDECT)–derived pulmonary perfusion maps for differentiation between acute pulmonary embolism (PE) and chronic thromboembolic pulmonary hypertension (CTEPH).

**Methods:**

This retrospective study included 131 patients (57 patients with acute PE, 52 CTEPH, 22 controls), who underwent CT pulmonary angiography on a dlDECT. Normal and malperfused areas of lung parenchyma were semiautomatically contoured using iodine density overlay (IDO) maps. First-order histogram features of normal and malperfused lung tissue were extracted. Iodine density (ID) was normalized to the mean pulmonary artery (MPA) and the left atrium (LA). Furthermore, morphological imaging features for both acute and chronic PE, as well as the combination of histogram and morphological imaging features, were evaluated.

**Results:**

In acute PE, normal perfused lung areas showed a higher mean and peak iodine uptake normalized to the MPA than in CTEPH (both *p* < 0.001). After normalizing mean ID in perfusion defects to the LA, patients with acute PE had a reduced average perfusion (ID_mean,LA_) compared to both CTEPH patients and controls (*p* < 0.001 for both). ID_mean,LA_ allowed for a differentiation between acute PE and CTEPH with moderate accuracy (AUC: 0.72, sensitivity 74%, specificity 64%), resulting in a PPV and NPV for CTEPH of 64% and 70%. Combining ID_mean,LA_ in the malperfused areas with the diameter of the MPA (MPA_dia_) significantly increased its ability to differentiate between acute PE and CTEPH (sole MPA_dia_: AUC: 0.76, 95%-CI: 0.68–0.85 vs. MPA_dia_ + 256.3 * ID_mean,LA_ − 40.0: AUC: 0.82, 95%-CI: 0.74–0.90,* p* = 0.04).

**Conclusion:**

dlDECT enables quantification and characterization of pulmonary perfusion patterns in acute PE and CTEPH. Although these lack precision when used as a standalone criterion, when combined with morphological CT parameters, they hold potential to enhance differentiation between the two diseases.

**Clinical relevance statement:**

Differentiating between acute PE and CTEPH based on morphological CT parameters is challenging, often leading to a delay in CTEPH diagnosis. By revealing distinct pulmonary perfusion patterns in both entities, dlDECT may facilitate timely diagnosis of CTEPH, ultimately improving clinical management.

**Key Points:**

*• Morphological imaging parameters derived from CT pulmonary angiography to distinguish between acute pulmonary embolism and chronic thromboembolic pulmonary hypertension lack diagnostic accuracy.*

*• Dual-layer dual-energy CT reveals different pulmonary perfusion patterns between acute pulmonary embolism and chronic thromboembolic pulmonary hypertension.*

*• The identified parameters yield potential to enable more timely identification of patients with chronic thromboembolic pulmonary hypertension.*

## Introduction

Acute pulmonary embolism (PE) is one of the main differential diagnoses in patients presenting with chest pain and/or dyspnea, ranking high among the most common causes of death from cardiovascular disease [1, 2]. Chronic thromboembolic pulmonary hypertension (CTEPH) is a rare but potentially fatal sequela of prior acute PE with incomplete recanalization or recurrent (sub-)clinical pulmonary emboli [3–6]. Clinically, CTEPH is defined by an increase in mean pulmonary artery pressure (mPAP) at rest > 20 mmHg, a pulmonary arterial wedge pressure < 15 mmHg, a mismatch on lung ventilation/perfusion (V/Q) scintigraphy, and/or specific diagnostic signs of chronic thromboembolism on angiography after ≥3 months of therapeutic anticoagulation [7, 8]. There is great uncertainty about the true incidence of CTEPH, which differs between ∼0.6% in “all-comers” and ∼3% in “PE-survivors” populations [6]. This can at least be partly attributed to the difficulties with identifying patients that already present with CTEPH at their PE index event [4, 9]. A variety of computed tomography pulmonary angiography (CTPA) parameters, which is the key imaging modality to rule out suspected PE [10], have been suggested to identify CTEPH [11–14]. These include direct vascular features such as laminated thrombi with obtuse angles to the contrast column, vessel narrowing or complete retraction, intimal irregularities, “webs and bands,” and post-stenotic dilatation. Indirect vascular features encompass dilatation of the mean pulmonary artery (MPA) or enlargement of bronchial collateral vessels. Indirect cardiac features, such as right ventricular hypertrophy, and parenchymal features, such as mosaic perfusion or parenchymal bands, are also suggestive for CTEPH. However, these imaging features are typically subtle and thus often initially overlooked [15], leading to a considerable delay in diagnosis [12].

By acquiring two spectrally distinct datasets, dual-energy CT (DECT) enables the computation of material-specific images. Based on the different absorption characteristics of iodine and the pulmonary parenchyma [16], DECT allows for a mapping of pulmonary iodine uptake, which is considered a surrogate indicator for pulmonary perfusion [17–19]. Characteristically, pulmonary perfusion abnormalities in CTEPH are patchy or multisegmental, sharply defined, wedge-shaped, and hypoattenuating [20, 21]. The latter is also described for acute PE [22]. For both entities, DECT has been proven as a feasible method for visualization and quantification of pulmonary perfusion defects [20, 22–28]. Furthermore, the generated iodine density overlay (IDO) maps improve diagnosis in acute PE and CTEPH [22, 29–31].

In CTEPH, pulmonary perfusion is maintained by the formation of systemic-to-pulmonary anastomoses and/or the dilatation of the vasa privata of the lung [32]. Earlier studies for two-phase and more recent studies for single-phase DECT suggest that DECT allows for a quantification of distinct differences in regional perfusion patterns between acute PE and CTEPH, which most likely can be ascribed to the maintained blood flow in malperfused lung areas in CTEPH via these systemic collaterals [33, 34]. Yet, both studies are limited primarily by their small sample sizes, and secondly due to the missing assessment of the diagnostic implications of their findings. Furthermore, two-phase DECT approaches hold the method’s inherent limitation of extra-radiation exposure.

Recently, we demonstrated the potential of single-phase dual-layer dual-energy (dlDECT) to semiautomatically detect and quantify pulmonary perfusion abnormalities in PH [30]. Given the diagnostic challenges of CTEPH, we therefore sought to quantitatively assess dlDECT-derived pulmonary perfusion in acute PE and CTEPH. Our objective was to evaluate its diagnostic ability to detect and differentiate acute and chronic stages of pulmonary thromboembolism.

## Materials and methods

### Study population

This study was approved by the local institutional review board (Ethics Committee of the Faculty of Medicine from the University of Cologne, Cologne, Germany). Necessity for informed consent was waived due to the retrospective design of the study. All clinical investigations were conducted in accordance with the Declaration of Helsinki.

This was a single-center, retrospective study. All patients underwent CTPA on dlDECT between June 2016 and February 2022, either due to suspected acute PE or CTEPH. Final diagnosis of CTEPH was reached by expert consensus based on right heart catheterization (RHC), V/Q scintigraphy, and further tests, given the retrospective study design, in accordance with the 2015 ESC/ERS guidelines [8]. Inclusion criteria for patients with acute PE were as follows: (1) suspicion of acute PE based on patient’s medical history and (2) concordant imaging findings on CTPA. Patients with (1) previous acute PE or known chronic thromboembolic disease (CTED) based on patient’s medical history or (2) direct vascular signs of chronicity (laminated thrombus with obtuse angle to the contrast column or calcification, intravascular webs, complete arterial occlusion, arterial narrowing or retraction, post-stenotic vascular dilatation [12]) or (3) non-thrombotic occlusion on CTPA were excluded from the acute PE group.

A total of 22 patients served as a control cohort. Among them, 14 had been clinically suspected of having PH, but were ruled out based on RHC results (mPAP < 25 mmHg) and showed no signs of CTED, as assessed by V/Q scintigraphy, CTPA, and pulmonary angiography when necessary. The remaining 8 patients underwent CTPA due to suspected acute PE but revealed no signs of acute or chronic PE upon assessment by a board-certified radiologist and presented no pulmonary comorbidities.

### Image acquisition and reconstruction

CT data were acquired on a dlDECT (IQon, Philips Healthcare). All patients received an intravenous 50 mL bolus of contrast media (300 mg iodine/mL, Accupaque, GE Healthcare) followed by a 40 mL NaCl chaser, both injected with a flow rate of 4 mL/s. After reaching an attenuation of 150 HU in the MPA, scanning in craniocaudal direction was initiated with a delay of 4.9 s. The acquisition parameters were as follows: slice collimation 64 × 0.625 mm; rotation time 0.33 s; tube potential 120 kV; tube current 75 mAs_ref_ with activated automatic tube current modulation. For all reconstructions, a dedicated spectral reconstruction algorithm with a soft tissue kernel was used (Spectral, B, Philips Healthcare). Images were reconstructed in axial orientation every 0.5 mm with a slice thickness of 1 mm. Matrix was set to 512 × 512. Both conventional images, identical to images reconstructed with the vendors hybrid-iterative reconstruction algorithm (iDose4, Philips Healthcare) [35], and IDO maps were reconstructed.

### Image analysis

#### Morphological CTPA analysis

##### **Assessment of morphological imaging features**

For all patients, a radiologist with 4 years of experience in cardiovascular imaging (R.J.G.) recorded direct vascular features, indirect vascular features, and parenchymal features associated with both acute and chronic PE, as detailed in Table 1 and reference [12].

**Table 1:** Morphological imaging features in acute PE, CTEPH, and controls

	APE	CTEPH	Controls	***p***
Parameter	(***n*** = 57)	(***n*** = 52)	(***n*** = 22)	
Direct vascular features^§^				
Acute thrombus morphology*		0/52 (0%)		
Intravascular webs		34/52 (65.4%)		
Complete arterial occlusion		16/52 (30.8%)		
Arterial narrowing or retraction		47/52 (90.4%)		
Post-stenotic vascular dilatation		11/52 (21.2%)		
Indirect vascular features				
MPA_dia_	28.8 ± 4.5	34.0 ± 5.5	26.7 ± 2.6	< 0.001 **
MPA_dia_/aorta_dia_ ratio	0.85 ± 0.14	1.05 ± 0.22	0.83 ± 0.10	**< 0.001 †**
RV/LV ratio^+^	1.09 ± 0.36	1.26 ± 0.43	0.93 ± 0.13	**0.02 ‡**
Flattening of the interventricular septum	18/57 (31.6%)	30/52 (57.7%)	0/22 (0%)	< 0.001
RV-hypertrophy^$^	1/57 (1.8%)	22/52 (42.3%)	0/22 (0%)	< 0.001
Diameter of the bronchial arteries, when detectable (31/46/5)	1.6 ± 0.31	2.4 ± 0.74	1.5 ± 0.16	**< 0.001 ||||**
Parenchymal features				
Mosaic perfusion	0/57 (0%)	16/52 (30.8%)	0/22 (0%)	< 0.001
Parenchymal bands	0/57 (0%)	21/52 (40.4%)	0/22 (0%)	< 0.001
Pulmonary infarction	9/57 (15.8%)	0/52 (0%)	0/22 (0%)	< 0.001

Continuous data are given as mean ± standard deviation. Categorical data are given as *n*/*n* (%)

^§^Not given for APE and controls as the presence of direct vascular features led to an exclusion from either the acute PE or the control group, respectively; *preserved caliber of the vessel; central (“polo mint” sign if imaged in short axis, or “railway track” sign if imaged in long axis) or eccentric filling defect; ^+^on a multiplanar-reformatted four-chamber view; ^$^characterized by free wall thickness >4 mm

^**^CTEPH vs. APE and CTEPH vs. controls,* p* < 0.001, respectively; APE vs. controls, *p = 0.12.* †CTEPH vs. APE and CTEPH vs. controls, *p* < 0.001, respectively; APE vs. controls, *p = 1.00*; ‡CTEPH vs. APE, *p* = 0.042; CTEPH vs. controls, *p* < 0.01; APE vs. controls, *p = 0.45*; ||||CTEPH vs. APE, *p* < 0.001; CTEPH vs. controls, *p* = 0.01; APE vs. controls, *p = 1.00*

*PE*, pulmonary embolism; *CTEPH*, chronic thromboembolic pulmonary hypertension; *APE*, acute pulmonary embolism; *MPA*_*dia*_, diameter mean pulmonary artery; *Aorta*_*dia*_, diameter aorta; *RV*, right ventricle; *LV*, left ventricle

##### **Assessment of thrombus/vascular occlusion level**

Thrombus levels in CTEPH have been shown to correlate with the degree of systemic collateral supply [36] and perfusion pattern [37, 38]. Therefore, thrombus/vascular occlusion level in CTEPH was assessed and classified as either central (cCTEPH) or peripheral (pCTEPH) as described previously [37]. In brief, applying Boyden’s nomenclature [39], cCTEPH was defined by the presence of chronic clots at the level of the pulmonary trunk and the main and lobar pulmonary arteries. pCTEPH was defined by the presence of CT features of chronic PE at the level of segmental and/or subsegmental arteries.

#### Lung segmentation and dlDECT-based lung perfusion analysis

Semiautomatic segmentation of the lung into normal and malperfused lung areas was achieved as previously described [30]. In brief, threshold segmentation was performed on automatically derived and manually verified lung volumes based on the IDO maps using a dedicated software solution for volumetric iodine quantification (ISD, ThresholdSegmentation (1.1), Philips IntelliSpace Release 11).

Regions of interest, accounting for at least 50% of the vascular/atrial area at the largest diameter, were manually drawn to assess the mean iodine density (ID) in the MPA and the left atrium (LA), respectively. These readouts were used to define three lung areas as follows: malperfused areas with an ID of less than 5% of the MPA, normal perfused areas with an ID of more than 5% of the MPA and less than 50% of the LA, and the vessel compartment with an ID of more than 50% of the LA.

#### Histogram analysis of lung perfusion and normalization of histogram parameters

Histogram analysis included the following first-order parameters for normal and malperfused lung compartments: ID_mean_, ID_max_, ID_kurtosis_, and ID_skewness_. Values for ID_mean_ and ID_max_ were normalized to the feeding vessel, the MPA (ID_mean,MPA_, ID_max,MPA_), as described previously [40]:

$${\mathrm{ID}}_{\mathrm{mean},\mathrm{MPA}}=\frac{\mathrm{meanID}}{\mathrm{meanIDMPA}}{\mathrm{ID}}_{\mathrm{max},\mathrm{MPA}}=\frac{\mathrm{maxID}}{\mathrm{meanIDMPA}}$$

In addition, to account for systemic collateral supply of the lung, values for ID_mean_ and ID_max_ were also normalized to the LA (ID_mean,LA_, ID_max,LA_):

$${\mathrm{ID}}_{\mathrm{mean},\mathrm{LA}}=\frac{\mathrm{meanID}}{\mathrm{meanIDLA}}{\mathrm{ID}}_{\mathrm{max},\mathrm{LA}}=\frac{\mathrm{maxID}}{\mathrm{meanIDLA}}$$

### Statistical analysis

The Shapiro-Wilk test was used to test for normality. Differences in continuous, parametric data were compared using the *t*-test. Continuous, independent, nonparametric data were compared using the Mann-Whitney *U* test. Differences in categorical data were identified using Pearson’s chi-squared test. To compare variances among and between the subgroups concerning continuous, parametric data, the ANOVA test was used. After assessing the equality of variances using Levene’s test, post hoc testing was performed with Bonferroni adjustment for multiple comparisons. Differences between the subgroups for categorical, nonparametric variables were assessed using the Kruskal-Wallis test and Dunn-Bonferroni corrected post hoc analysis.

To assess the diagnostic performance of the derived histogram parameters, the respective patient subcohorts (subcohort 1: acute PE/CTEPH vs. control group, subcohort 2: acute PE vs. CTEPH) were split into a training and a validation set (80/20 split) using a freeware research data randomizer (https://www.randomizer.org). Diagnostic performance was evaluated by calculating the area under the receiver operating characteristic curve (AUC) and positive and negative predictive value (PPV, NPV). Cut-off values for optimal sensitivity and specificity were calculated using Youden’s index. Only the histogram features with the best diagnostic performance based on AUC analyses are reported in the “Results” section.

To examine whether histogram parameters provide additional information to discriminate acute PE from CTEPH, histogram parameters and morphological imaging features, which were not a priori included in the exclusion criteria for acute PE, were combined using the method introduced by Pepe et al [41]. Differences between AUCs were evaluated using the DeLong test [42].

A *p*-value of < 0.05 was considered statistically significant. Statistical analysis was performed using SPSS software (IBM SPSS Statistics for macOS, Version 27.0) and R (R Core Development Team, version 4.2.0), using RStudio (RStudio, Version 2023.03.1).

## Results

### Patient demographics

**Fig. 1:**
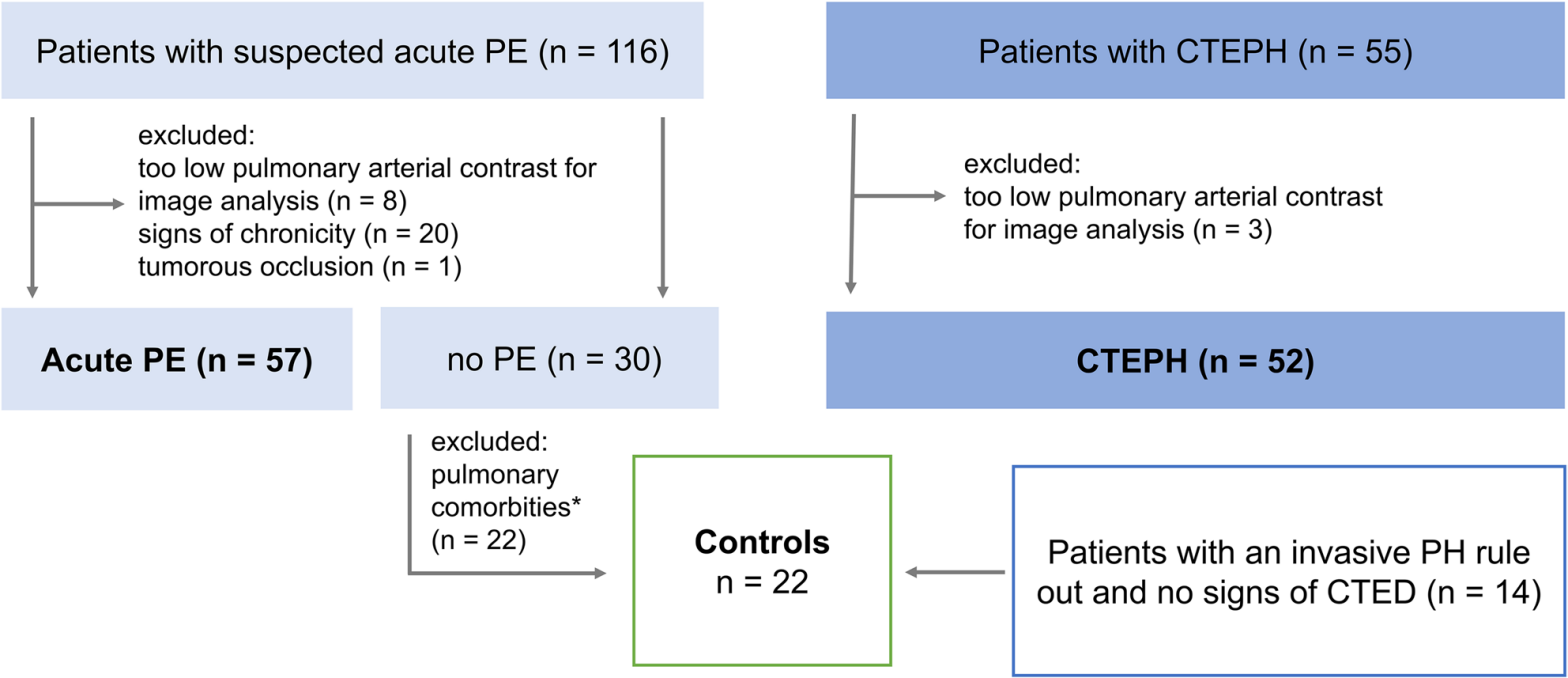
Study flow chart. PE, pulmonary embolism; CTEPH, chronic thromboembolic pulmonary hypertension; CTED, chronic thromboembolic disease. *For example, pleural effusion, pneumonia, pulmonary oncologic manifestations. Pulmonary fibrosis and emphysema were not exclusion criteria

A total of 116 patients with suspected acute PE and 55 patients with CTEPH were included. Of these 171 patients, 32 were excluded: 11 patients due to too low intraarterial contrast (mean attenuation in the MPA: 261.1 ± 4.9 HU in included scans vs. 217.3 ± 19.5 HU in excluded scans, *p* < 0.001), 20 patients from the acute PE arm with subacute PE based on patient´s medical history or imaging features, and one patient with a tumorous vascular occlusion (Fig. 1). There were no differences between the groups regarding age (mean ± SD: acute PE, 61 ± 16 years; CTEPH, 62 ± 16 years; controls, 63 ± 18 years; *p* = 0.76). While gender distribution between the acute PE (m/f, 32/25) and the CTEPH group (m/f, 24/28) was comparable (*p* = 0.30), controls were more often female (m/f, 5/17) as compared to PE patients (*p* = 0.008). Percentages of normal perfused and malperfused areas in the lung were 68.6 ± 17.9% and 28.3 ± 18.4%, respectively, for patients with acute PE, 53.6 ± 20.0% and 44.1 ± 20.2%, respectively, for patients with CTEPH, and 73.1 ± 18.2% and 23.9 ± 18.5%, respectively, for controls. There was no difference between groups regarding the number of beam hardening artifacts due to contrast in the subclavian vein and/or extracorporeal foreign material (acute PE, 30/57; CTEPH, 32/52; controls, 14/22; *p* = 0.54). Manual editing of the generated lung volumes was required in 54 of 131 cases (41.2%) and more frequently necessary in acute PE compared to both other groups (acute PE, 37/57; CTEPH, 15/52; controls, 2/22; *p* < 0.001).

### Morphological imaging features

Morphological imaging features are displayed in Table 1. Compared to patients with acute PE and controls, those with CTEPH had a greater MPA diameter (acute PE, 28.8 ± 4.5; CTEPH, 34.0 ± 5.5; controls, 26.7 ± 2.6; *p* < 0.001), a higher MPA/aorta ratio (acute PE, 0.85 ± 0.14; CTEPH, 1.05 ± 0.22; controls, 0.83 ± 0.10; *p* < 0.001), and a higher right ventricle (RV) to left ventricle (LV) diameter ratio (acute PE, 1.09 ± 0.36; CTEPH, 1.26 ± 0.43; controls, 0.93 ± 0.13; *p* = 0.02), as well as larger diameters of the bronchial arteries (acute PE, 1.6 ± 0.31; CTEPH, 2.4 ± 0.74; controls, 1.5 ± 0.16;* p* < 0.001). Furthermore, a flattened interventricular septum and right ventricular hypertrophy were observed more frequently in the CTEPH group compared to the other two groups (*p* for both < 0.001; Table 1).

### dlDECT-derived pulmonary perfusion

#### Differentiation between controls and patients with thromboembolic disease

**Fig. 2:**
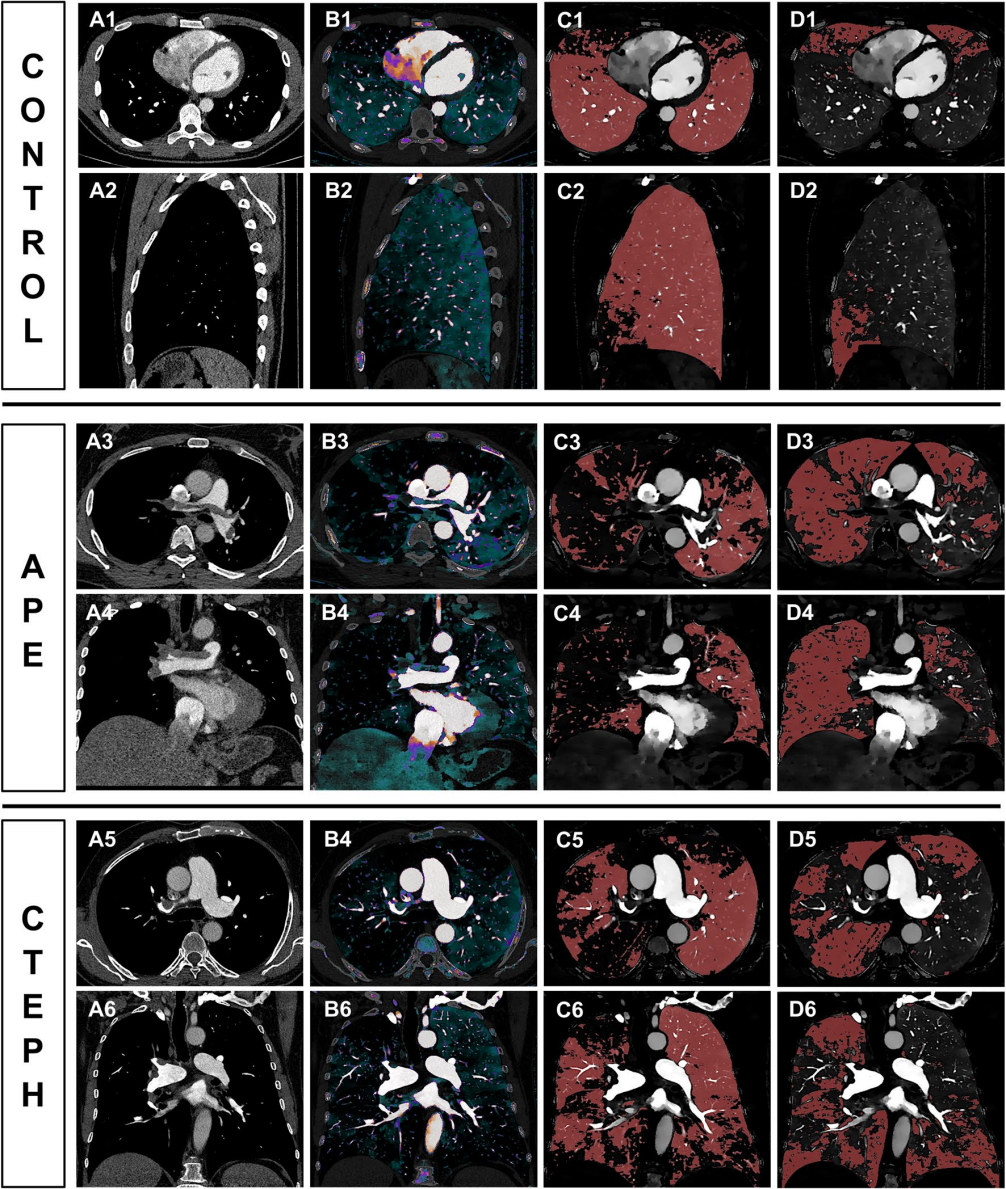
Conventional reconstructions (**A1–A6**), corresponding IDO images (**B1–B6**), and automatically derived normal (**C1–C6**) and malperfused lung areas (**D1–D6**) illustrating the physiological ventro-dorsal gradient of pulmonary blood volume in the supine patient as well as the visually similar perfusion patterns in acute PE (middle) and CTEPH (bottom). IDO, iodine density overlay; APE, acute pulmonary embolism; CTEPH, chronic thromboembolic pulmonary hypertension

**Fig. 3:**
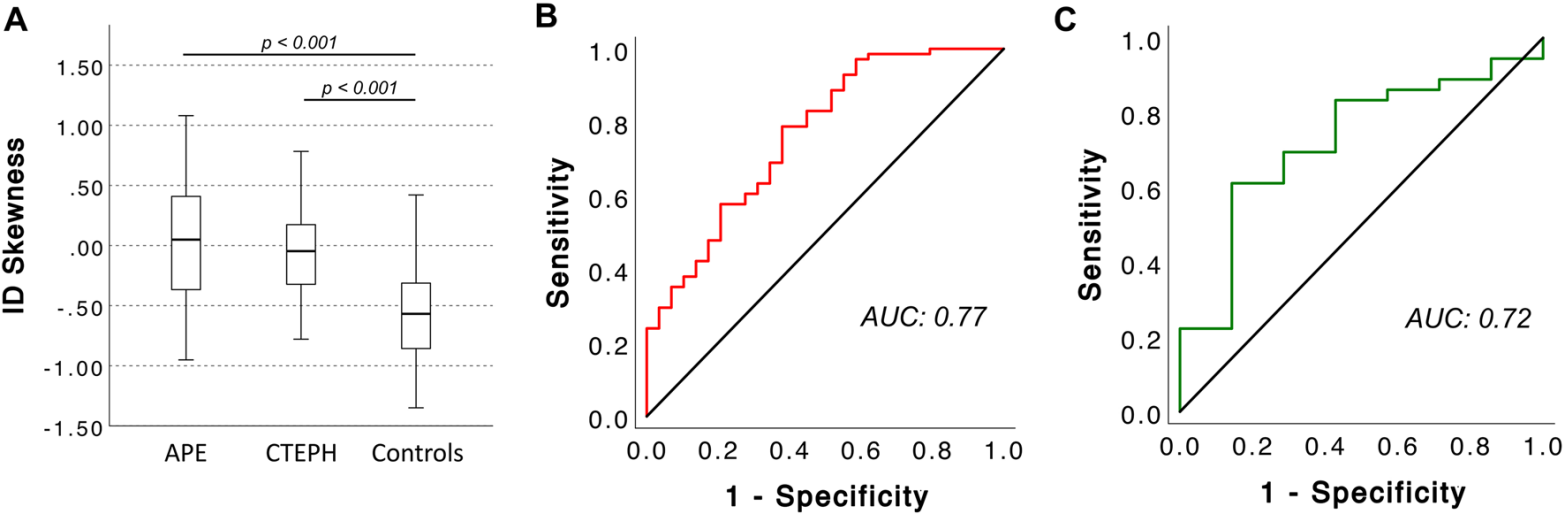
Box (25th percentile, median, and 75th percentile) and whisker (10th and 90th percentile) plots for ID_skewness_ in malperfused lung areas (**A**) and diagnostic accuracy of ID_skewness_ in malperfused lung areas for acute PE/CTEPH based on AUC analysis in the training dataset (**B**) and the test dataset (**C**). ID, iodine density; AUC, area under curve; APE, acute pulmonary embolism; CTEPH, chronic thromboembolic pulmonary hypertension

   Figure 2 exemplifies the semiautomatically derived normal and malperfused lung areas in a control, a patient with acute PE, and a patient with CTEPH. Patients with acute PE had a significantly lower ID in the MPA as compared to controls (*p* = 0.046). Right-to-left-heart contrast transit *via* the pulmonary vascular bed, as indicated by the ID in the LA, revealed no differences between groups based on post hoc analysis. Malperfused lung areas in acute PE and CTEPH were perfused less when being standardized to the MPA (ID_mean,MPA_) and more homogenous (ID_skewness_) in comparison to controls (ID_mean,MPA_: acute PE, 0.022 ± 0.005; CTEPH, 0.023 ± 0.004; controls, 0.028 ± 0.005; ID_skewness_: acute PE, 0.03 ± 0.48; CTEPH, −0.06 ± 0.38; controls, −0.57 ± 0.45;* p* for all < 0.001; Table 2 and Fig. 3).

**Table 2:** dlDECT-based pulmonary perfusion characteristics in acute PE, CTEPH, and controls

	APE	CTEPH	Controls	*p*	APE vs. CTEPH	APE vs. controls	CTEPH vs. controls
Parameter	(*n* = 57)	(*n* = 52)	(*n* = 22)		*p*	*p*	*p*
Feeding vessel							
ID_MPA_ (mg/mL)	12.6 ± 5.2	14.3 ± 3.9	15.3 ± 3.8	**0.025**	0.13	** 0.046**	1.00
ID_LA_ (mg/mL)	10.1 ± 2.8	9.0 ± 2.3	10.4 ± 2.9	**0.049**	0.11	1.00	0.13
Normal perfused lung							
% normal perfused lung	68.6 ± 17.9	53.6 ± 20.0	73.1 ± 18.2	**< 0.001**	**< 0.001**	0.99	**< 0.001**
ID_mean,MPA_	0.13 ± 0.04	0.10 ± 0.02	0.11 ± 0.03	**< 0.001**	**< 0.001**	0.05	1.00
ID_mean,LA_	0.15 ± 0.03	0.16 ± 0.04	0.16 ± 0.05	0.17			
ID_max,MPA_	0.44 ± 0.16	0.33 ± 0.13	0.35 ± 0.09	**< 0.001**	**< 0.001**	0.07	0.75
ID_max,LA_	0.4993 ± 0.0035	0.4998 ± 0.0003	0.4990 ± 0.0042	**0.04**	0.97	0.19	** 0.031**
ID_kurtosis_	5.4 ± 3.5	5.3 ± 3.6	6.3 ± 3.9	0.45			
ID_skewness_	2.0 ± 0.7	2.0 ± 0.7	2.1 ± 0.8	0.85			
Malperfused lung							
% malperfused lung	28.3 ± 18.4	44.1 ± 20.2	23.9 ± 18.5	**< 0.001**	**< 0.01**	1.00	** < 0.01**
ID_mean,MPA_	0.022 ± 0.005	0.023 ± 0.004	0.028 ± 0.005	**< 0.001**	1.00	**< 0.001**	** < 0.001**
ID_mean,LA_	0.028 ± 0.012	0.040 ± 0.016	0.046 ± 0.022	**< 0.001**	**< 0.001**	**< 0.001**	0.70
ID_max,MPA_	0.0497 ± 0.0003	0.0498 ± 0.0002	0.0499 ± 0.0001	0.07			
ID_max,LA_	0.06 ± 0.02	0.09 ± 0.03	0.08 ± 0.04	**< 0.001**	**< 0.001**	0.06	0.80
ID_kurtosis_	−1.1 ± 0.3	−1.1 ± 0.2	−0.7 ± 0.8	**0.04**	0.26	0.05	0.86
ID_skewness_	0.03 ± 0.48	−0.06 ± 0.38	−0.57 ± 0.45	**< 0.001**	1.00	**< 0.001**	** < 0.001**

Data are given as mean ± standard deviation. *n.s.*, not significant

*dlDECT*, dual-layer dual-energy CT; *PE*, pulmonary embolism; *CTEPH*, chronic thromboembolic pulmonary hypertension; *APE*, acute pulmonary embolism; *ID*, iodine density; *MPA*, mean pulmonary artery; *LA*, left atrium

On the basis of AUC analysis, ID_skewness_ in malperfused lung areas enabled for identification of acute PE/CTEPH (training set: AUC 0.77, 95%-CI: 0.66–0.87; validation set: AUC 0.72, 95%-CI: 0.53–0.92; Fig. 3). Applying a cut-off of −0.35 as determined by Youden’s index resulted in a sensitivity of 79% and a specificity of 62% as well as a positive predictive value (PPV) of 90% and a negative predictive value (NPV) of 29% in the validation dataset.

#### Differentiation between acute PE and CTEPH

**Fig. 4:**
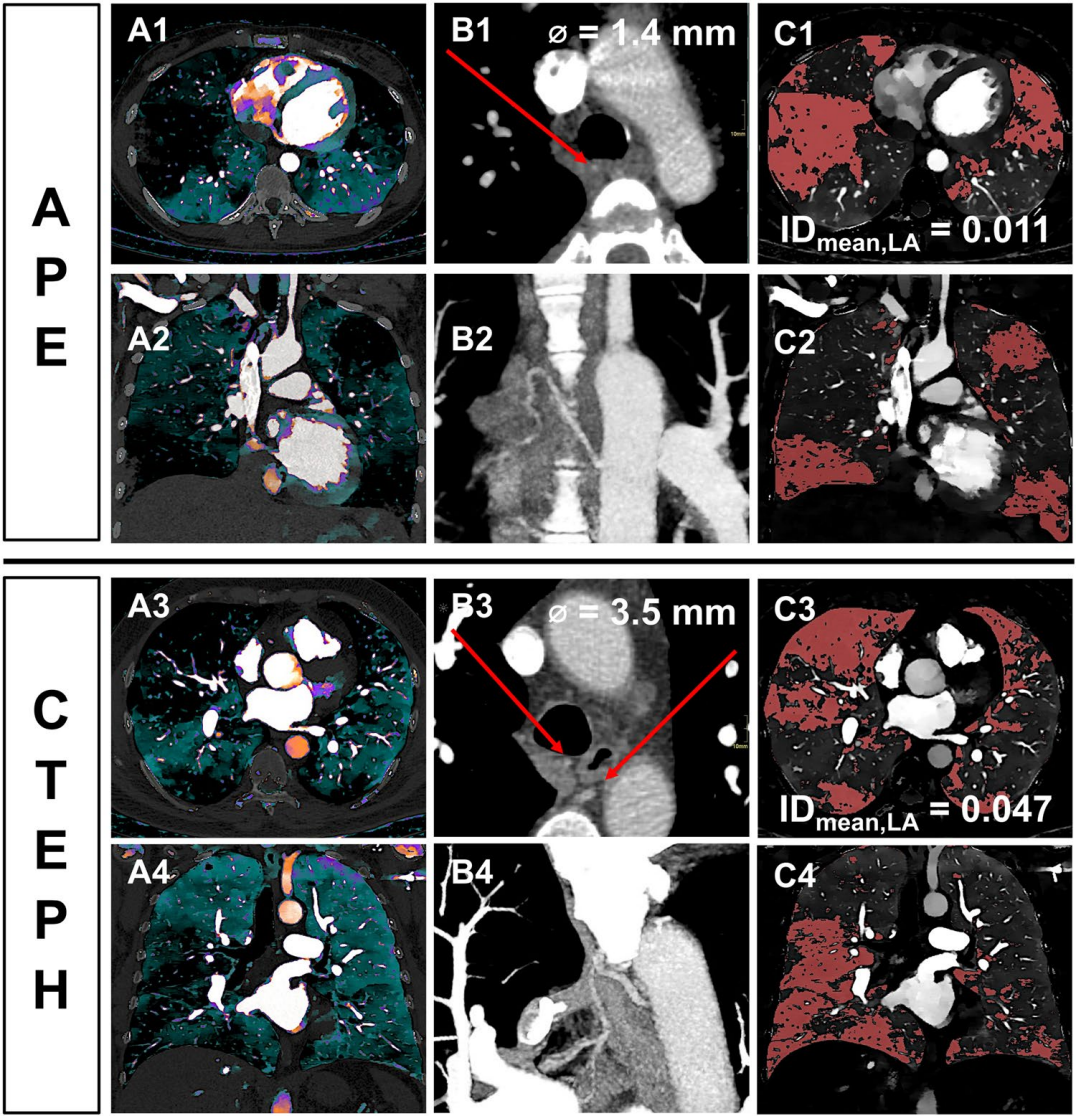
dlDECT-based assessment of pulmonary perfusion via systemic collaterals in a patient with acute PE (top) and a patient suffering from CTEPH (bottom). Axial and paracoronar multiplanar reconstructions show the enlarged bronchial arteries (>1.5 mm) in the CTEPH patient (**B3** and **B4**) leading to an increased perfusion in embolic lung areas as indicated by an increased ID_mean,LA_ (**C1**/2 vs. **C3**/4). APE, acute pulmonary embolism; CTEPH, chronic thromboembolic pulmonary hypertension; ID, iodine density; LA, left atrium

**Fig. 5:**
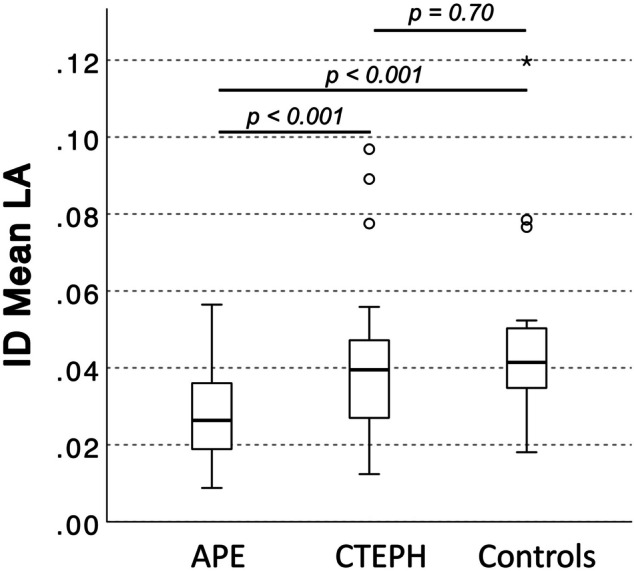
Box (25th percentile, median, and 75th percentile) and whisker (10th and 90th percentile) plots of ID_mean,LA_ in malperfused lung areas. ID, iodine density; LA, left atrium; APE, acute pulmonary embolism; CTEPH, chronic thromboembolic pulmonary hypertension

In acute PE, normal perfused lung areas took up more iodine on average than in CTEPH when being normalized to the MPA (ID_mean,MPA_: 0.13 ± 0.04 vs. 0.10 ± 0.02,* p* < 0.001). Normalizing mean iodine uptake in perfusion defects to the LA patients with acute PE showed a reduced perfusion compared to CTEPH patients and controls (ID_mean,LA_, both *p* < 0.001; Figs. 4 and Fig. 5).

There were no differences for ID_mean,MPA_ and ID_skewness_ in malperfused lung areas between acute PE, cCTEPH, and pCTEPH respectively (Table 3). Furthermore, ID_mean,LA_ in CTEPH was similar with regard to thrombus/vascular occlusion level.

**Table 3:** dlDECT-based perfusion characteristics in malperfused lung areas in acute PE and central and peripheral CTEPH

Parameter	APE (*n* = 57)	cCTEPH (*n* = 14)	pCTEPH (*n* = 38)	*p*
Malperfused lung				
ID_mean,MPA_	0.022 ± 0.005	0.021 ± 0.003	0.024 ± 0.003	0.04 *
ID_mean,LA_	0.028 ± 0.012	0.035 ± 0.019	0.041 ± 0.016	< 0.001 **
ID_skewness_	0.03 ± 0.48	0.14 ± 0.29	−0.13 ± 0.39	0.06

Data are given as mean ± standard deviation

^*^APE vs. cCTEPH, *p = 0.85*; APE vs. pCTEPH, *p = 0.17*; cCTEPH vs. pCTEPH, *p = 0.63*.  **APE vs. pCTEPH, *p* < 0.001; APE vs. cCTEPH, *p = 0.55*; cCTEPH vs. pCTEPH, *p = 0.34*

*dlDECT*, dual-layer dual-energy CT; *PE*, pulmonary embolism; *CTEPH*, chronic thromboembolic pulmonary hypertension; *APE*, acute pulmonary embolism; *cCTEPH*, central chronic thromboembolic pulmonary hypertension; *pCTEPH*, peripheral chronic thromboembolic pulmonary hypertension; *ID*, iodine density; *MPA*, mean pulmonary artery; *LA*, left atrium

ID_mean,LA_ in the malperfused areas allowed for a differentiation between acute PE and CTEPH with moderate accuracy based on ROC analysis (training dataset: AUC: 0.72, 95%-CI: 0.62–0.83; validation dataset: AUC: 0.71, 95%-CI: 0.47–0.95). A cut-off value of 0.031 resulted in a PPV and NPV for CTEPH of 64% and 70% in the validation dataset.

Combining ID_mean,LA_ in the malperfused areas with the diameter of the MPA (MPA_dia_) significantly increased the ability to differentiate between acute PE and CTEPH (sole MPA_dia_ evaluation: AUC: 0.76, 95%-CI: 0.68–0.85 vs. MPA_dia_ + 256.3 * ID_mean,LA_ − 40.0: AUC: 0.82, 95%-CI: 0.74–0.90, *p* = 0.04). Furthermore, combining ID_mean,LA_ in the malperfused areas with the diameter of the bronchial arteries, there was a trend to an increase in the AUC (sole bronchial artery diameter evaluation: AUC: 0.85, 95%-CI: 0.77–0.94 vs. bronchial artery diameter + 33.11 * ID_mean,LA_ − 3.15: AUC: 0.90, 95%-CI: 0.84–0.97, *p* = 0.08).

## Discussion

To the best of our knowledge, this study reports data from the largest cohort of acute PE and CTEPH patients that were characterized by DECT. Several notable findings can be reported: First, perfusion deficit patterns in acute PE and CTEPH can be assessed and quantified by dlDECT without additional radiation exposure. Second, ID_skewness_ in malperfused lung areas can differentiate between patients with thromboembolic perfusion defects and controls. Third, patients with acute PE show an overperfusion in non-embolic lung areas. Fourth, dlDECT allows for a quantification of iodine uptake in malperfused lung areas relative to systemic-to-pulmonary collaterals unveiling distinct perfusion patterns in acute PE and CTEPH. Lastly, combining dlDECT-based pulmonary perfusion with morphological CT parameters increases the diagnostic accuracy in differentiating between acute PE and CTEPH.

DECT has proven applicability in acute PE and CTEPH to visualize pulmonary perfusion defects [20, 22–28]. Characteristically, in acute PE as well as in CTEPH, these are described as sharply defined, wedge-shaped, and hypoattenuating [20–22]. We demonstrated that the visual similarities in perfusion deficit patterns, shared by both entities, can be assessed and quantified by a low ID_mean,MPA_ and an ID_skewness_ close to zero in malperfused lung areas. Giordano et al demonstrated that in pCTEPH PE-like perfusion defects are present in only 37.5%, while most patients with pCTEPH show a patchy perfusion pattern [38]. Concordantly, based on ID_mean,MPA_ and ID_skewness_ in malperfused lung areas, we did see a trend towards a more PE-like perfusion pattern in central than in pCTEPH.

The diagnostic implications of DECT-based pulmonary perfusion maps have been excessively investigated for acute PE [22–24, 31, 43, 44] and to a lesser degree for CTEPH [27, 29, 30, 45]. Noteworthy, controls revealed a considerable amount of malperfused lung areas which did not differ compared to patients with acute PE. This likely reflects the physiological ventro-dorsal gradient of pulmonary blood volume in the supine patient [46, 47]. Moreover, given the study’s retrospective design, PH exclusion was based on the 2015 ESC guidelines, employing an mPAP cut-off of 25 mmHg [10]. Yet, according to the recently updated guidelines [8], PH is defined by an invasively measured mPAP at rest > 20 mmHg. Under this criterion, only 7 of the 14 patients from the control group with a clinical suspicion of PH would be cleared of PH, with an additional 3 showing a borderline mPAP of 19 or 20 mmHg. Given the known association between PH—irrespective of its etiology—and pulmonary perfusion abnormalities [27, 29, 30, 38], it seems plausible that the hemodynamic characteristics of our control group influenced our results to some degree. Consequently, the amount of malperfused lung areas offers limited diagnostic insight.

In contrast, ID_mean,MPA_ and ID_skewness_ in malperfused lung areas did not only differ between controls and acute PE/CTEPH patients but also allowed for the prediction of acute PE/CTEPH with considerable accuracy (PPV = 90%). However, the overall diagnostic performance of the semiautomatic dlDECT-derived parameters was lower than for most hitherto reported manual reading approaches [23, 28, 43]. Moreover, given an NPV of 29% for ID_skewness_ in malperfused lung areas, applying the reported cut-offs would result in a high rate of false negatives hampering their clinical implementation. Notwithstanding, it is important to note that it was beyond the scope of this study to develop a semi-automatic screening tool for acute PE/CTEPH. Rather, this study was intended as a proof-of-concept, which is why we reported the cut-offs according to Youden’s index. For their clinical implementation, these cut-offs would require adjustment depending on the clinical question at hand (i.e., rule-in vs. rule-out). Therefore, additional studies are warranted to identify the optimal cut-off values based on specific clinical requirements.

Numerous studies demonstrated promising results for computer-aided detection (CAD) or artificial intelligence (AI)–based detection of intravascular thrombi [48–50]. Taking our findings into account, a DECT approach integrating the conventional-based vascular and the IDO-based perfusion information might thus hold potential to outperform CAD/AI algorithms solely relying on conventional images.

Normal perfused lung areas in patients with acute PE stood out due to a higher perfusion compared to CTEPH patients, also revealing a trend to higher perfusion compared to controls. These findings indicate that the redistribution of the pulmonary blood flow in acute PE, which leads to a relative overperfusion of non-embolic regions [51, 52], can be quantified by dlDECT.

Pulmonary blood flow in CTEPH is commonly maintained via bronchopulmonary collaterals, which can account for up to 30% of the pulmonary blood flow [32]. In their two-phase DECT study, Hong et al elegantly demonstrated that this potentially explains the significantly higher enhancement of malperfused lung segments in delayed-phase images of CTEPH patients as compared to acute PE patients [33]. However, routine performance of this dual-phase DECT approach in patients suspected of acute PE or CTEPH seems hampered due to the additional X-ray irradiation for the delayed-phase image and the second image with a different acceleration voltage, respectively [6]. On the contrary, the ability to identify characteristic differences in the perfusion without additional radiation exposure may make dlDECT also applicable for routine practice in these patients. Furthermore, the aforementioned study by Hong et al was limited due to its small sample size and did not evaluate whether its findings influence the diagnostic accuracy in differentiating between acute PE and CTEPH. In contrast, we could demonstrate that the identified dlDECT parameters do not only enable differentiation between these two entities but also increase the diagnostic abilities of morphological CT parameters in distinguishing acute and chronic stages of pulmonary thromboembolism.

In contrast to dlDECT, V/Q scintigraphy, the current gold standard to rule out CTEPH [10], neither allows for a morphological nor for a functional assessment of systemic collaterals, because ^99m^Tc-maggroaggregatred albumin is trapped in the pulmonary capillary bed. As there is evidence that the degree of systemic collateral formation correlates with postsurgical outcome in CTEPH [53], dlDECT might thus be advantageous compared to V/Q scintigraphy in preoperative imaging.

## Limitations

Besides its retrospective design, this study has several limitations. First, the control group did not comprise truly healthy individuals but rather patients with either the clinical suspicion of PH or acute PE. Even more, applying the recently revised hemodynamic definition of PH [8], seven out of the control group patients were to be diagnosed with PH. Additionally, three patients exhibited a borderline mPAP of 19 or 20 mmHg. However, considering the retrospective design of our study, this limitation seems inevitable. In this context, it is important to highlight that, as of now, no reference values for DECT-based pulmonary perfusion exist. Future studies aiming at establishing these standard values are therefore of paramount importance. Second, the diagnostic performance to assess pulmonary perfusion was not evaluated against a reference standard, such as V/Q scintigraphy. Third, the semiautomatic lung segmentation approach did not allow for a differentiation between true perfusion and pseudodefects, e.g., due to beam hardening or motion artifacts [54]. Beam hardening artifacts represent a common problem in the DECT-based assessment of pulmonary perfusion [47], which is reflected by our results. However, beam hardening artifacts were equally frequent across all groups. Noteworthy, as opposed to earlier studies, we did not exclude patients with coexisting parenchymal lung disease, which is known to mimic CTEPH perfusion defects [30]. Fourth, there was a considerable selection bias, as patients with history or imaging features of subacute PE were excluded from the acute PE arm. This patient group potentially causes the biggest diagnostic uncertainties. Therefore, future studies on the perfusion characteristics in these patients are highly warranted. Fifth, we did not assess diagnostic or prognostic implications of our findings, e.g., whether the identified parameters affect diagnostic confidence or correlate with patient outcome. These questions should consequently be addressed in future studies. Lastly, our findings regarding the diagnostic accuracy of the diameter of the bronchial arteries need to be interpreted with caution as their quantitative evaluation was only feasible in a subset of patients due to scan timing or too small vessel diameter.

## Conclusion

Acute PE and CTEPH show different pulmonary perfusion patterns that can be semiautomatically assessed and quantified by single-phase dlDECT. These perfusion characteristics, potentially unveiling the different degrees of perfusion through systemic collaterals in both entities, increase diagnostic accuracy of morphological CT parameters for the differentiation between the two diseases without the need for additional radiation exposure.

## Abbreviations

AI: Artificial intelligence
AUC: Area under curve
CAD: Computer-aided detection
CTED: Chronic thromboembolic disease
CTEPH: Chronic thromboembolic pulmonary hypertension
cCTEPH: Central chronic thromboembolic pulmonary hypertension
DECT: Dual-energy CT
dlDECT: Dual-layer dual-energy CT
ID: Iodine density
IDO: Iodine density overlay
LA: Left atrium
MPA: Mean pulmonary artery
mPAP: Mean pulmonary artery pressure
NPV: Negative predictive value
pCTEPH: Peripheral chronic thromboembolic pulmonary
PE: Pulmonary embolism
PH: Pulmonary hypertension
PPV: Positive predictive value
RHC: Right heart catheterization
V/Q scintigraphy: Ventilation/perfusion scintigraphy

## Declarations

### Guarantor

The scientific guarantor of this publication is Roman J. Gertz.

### Statistics and biometry

One of the authors (MP) has significant statistical expertise.

### Study subjects or cohorts overlap

Some study subjects or cohorts have been previously reported in:

Gertz RJ, Gerhardt F, Kröger JR, Shahzad R, Caldeira L, Kottlors J, Große Hokamp N, Maintz D, Rosenkranz S, Bunck AC. Spectral detector CT-derived pulmonary perfusion maps and pulmonary parenchyma characteristics for the semiautomated classification of pulmonary hypertension. Front Cardiovasc Med. 2022 Feb 28;9:835732. https://doi.org/10.3389/fcvm.2022.835732. PMID: 35391852; PMCID: PMC8982082.

### Methodology

• retrospective

• diagnostic or prognostic study

• performed at one institution
